# Absolutely minimal semi–Lipschitz extensions

**DOI:** 10.1007/s00526-025-03169-1

**Published:** 2025-10-15

**Authors:** Aris Daniilidis, Trí Minh Lê, Francisco M. Venegas

**Affiliations:** 1 Institut für Stochastik und Wirtschaftsmathematik, VADOR E105-04 TU Wien, Wiedner Hauptstraße 8, A-1040 Wien, Austria; 2 Instituto de Ciencias de la Ingeniería, Universidad de O’Higgins, Rancagua, Chile

**Keywords:** Primary 26A16, 39B82, Secondary 35B50, 41A05

## Abstract

The notion of quasi-metric space arises by revoking the symmetry from the definition of a distance. Semi-Lipschitz functions appear naturally as morphisms associated with the new structure. In this work, under suitable assumptions on the quasi-metric space (analogous to standard ones in the metric case), we establish existence of optimal (that is, absolutely minimal) extensions of real-valued semi-Lipschitz functions from a subset of the space to the whole space. This is done in two different ways: first, by adapting the Perron method from the classical setting to this asymmetric case, and second, by means of an iteration scheme for (an unbalanced version of) the tug-of-war game, initiating the algorithm from a McShane extension. This new iteration scheme provides, even in the symmetric case of a metric space, a constructive way of establishing existence of absolutely minimal Lipschitz extensions of real-valued Lipschitz functions.

## Introduction

The classical problem of extending a Lipschitz function, preserving its Lipschitz constant, was initiated by McShane [29] and Whitney [37, footnote p.63]. Given a nonempty compact set $\Omega \subset \mathbb {R}^{n}$ and a Lipschitz function $f:\partial \Omega \rightarrow \mathbb {R}$ Aronsson [5] considered the problem of finding the “best” possible Lipschitz extension and established the following result:

- there exists a (unique) Lipschitz function $u:\Omega \rightarrow \mathbb {R}$ such that
  1.1 $$\begin{aligned} u|_{\partial \Omega }=f\qquad \text {and\qquad }\textrm{Lip}(u,\Omega )=\textrm{Lip}(f,\partial \Omega ) \end{aligned}$$
  with the additional property that
  1.2 $$\begin{aligned} \textrm{Lip}(u,V)=\textrm{Lip}(u,\partial V),\quad \text { for every open set }V\subset \subset \Omega . \end{aligned}$$

The classical notation $V\subset \subset \Omega$ means, as usual, that the closure $\overline{V}$ of the open set *V* is contained in $\Omega$. For any nonempty set $A\subset \mathbb {R}^{n}$, the Lipschitz constant $\textrm{Lip}(u,A)$ of *u* on *A* is defined by the formula

$$ \textrm{Lip}(u,A)=\sup _{x,y\in A{,}\;x\ne y}\dfrac{|u(x)-u(y)|}{|x-y|}. $$

Every function *u* satisfying (1.1) is called a *minimal Lipschitz extension* of *f*. If in addition *u* satisfies (1.2) then it is called *absolutely minimal Lipschitz extension* (AMLE, for short) of *f*.

AMLEs have been studied from many different perspectives, such as absolute minimizers of $L^{\infty }$ functionals, viscosity solutions of the infinity Laplace equation $\Delta _{\infty }u=0$ and value functions of a two-person tug-of-war game, see [2–4, 6, 14, 20, 35] and have several applications, for instance, in image processing and in studying the possible shape of a sandpile, see [10, 18]. Due to the relevance of AMLEs, over the last decades, there has been a growing effort to study this kind of functions in more general settings, for example, in separable length spaces and in metric measure spaces, see [17, 21, 22].

As a common phenomenon that often occurs with elliptic equations in Euclidean spaces, absolutely minimal Lipschitz functions exhibit an asymptotic mean value property. More precisely, a function *u* is absolutely minimal Lipschitz if and only if the following asymptotic expansion holds true in a viscosity sense ([26])

1.3 $$\begin{aligned} u(x)=\underbrace{\dfrac{1}{2}\left( \max _{y\in \overline{B(x,\varepsilon )}}u(y)+\min _{y\in \overline{B(x,\varepsilon )}}u(y)\right) }_{T_{\varepsilon } [u](x)}+~o(\varepsilon ^{2}),\quad \text { as }\varepsilon \rightarrow 0^{+}. \end{aligned}$$

Notice that the maximum and minimum in (1.3) are attained since we are considering Euclidean spaces. Let us mention that the above (symmetric) tug-of-war operator $u\mapsto T_{\varepsilon }[u]$, $\varepsilon >0$ has also been considered in [23, 24] for compact convex metric spaces to treat the problem of absolutely minimal extensions in with respect to a modulus of continuity $\omega$. Such extensions are defined in a similar way to AMLE functions by replacing the Lipschitz constant by a modulus of continuity. The operator $T_{\varepsilon }[u]$ averages, at every $x\in X$, the maximum and the minimum value of the function *u* on the ball $B(x,\varepsilon )$ and fixed points of the operator can be interpreted as the value function of a two–person tug–of–war game, see [35]. The sequence $\{u_{\varepsilon _{n}}\}_{n\ge 1}$ of fixed points of the operators $T_{\varepsilon _{n}}$ converges as $\varepsilon _{n}\rightarrow 0^{+}$ to an absolutely minimal extension with respect to the modulus of continuity $\omega$, see [23, 24].

Back to the Lipschitz case, a fixed point $u_{\varepsilon }$ of the operator $T_{\varepsilon }$ supplies a good approximation of an AMLE function (provided we know this latter exists). It is also an absolutely minimal Lipschitz function with respect to a new distance that depends on the initial distance and $\varepsilon >0$, see [28]. However, $u_{\varepsilon }$ is not an AMLE for the initial distance and it is also unclear if the passage to the limit, as $\varepsilon \rightarrow 0$ provides a function with this desired property. Therefore, as explicitly stated in [23, Remark 2.2], the above method does not give a formal proof of the existence of AMLE functions. In particular, to the best of our knowledge, no explicit tug-of-war constructive scheme leading to an AMLE function is known up-to-date.

In this manuscript we deal with quasi-metric spaces (Definition 1.1), that is, spaces equipped with an asymmetric distance, and study an analogue to AMLE optimal extension for the class of semi-Lipschitz functions (under analogous assumptions on the quasi-metric space as the ones usually employed in the metric case). These latter functions are naturally associated with the asymmetric structure of a quasi-metric space and consist of suitable alternatives to Lipschitz functions, see [12, 15, 16]. Therefore, the central notion of this work is the notion of *absolutely minimal semi-Lipschitz extension* (AMSL extension, for short), see Definition 2.3.

Studying AMLEs in the asymmetric setting has recently attracted a lot of interest since it arises while considering irreversible Finsler manifolds, see [1]. Furthermore, an asymmetric distance appears naturally while studying absolute minimizers of $L^{\infty }$ functionals with general Hamiltonians, see [11]. The PDE aspect of AMSL extensions has been extensively studied. Specifically, an AMSL extension can be equivalently viewed as a viscosity solution of the so-called Finsler $\infty$-Laplace equation. Based on this relation with PDEs, under mild smoothness assumptions on the norm, existence and uniqueness of AMSL in bounded domains of $\mathbb {R}^{n}$ have been obtained, see [30, 31]. An analog of (1.3) in the asymmetric framework has been observed in [32]. Concerning the regularity of planar AMLEs, they are $\mathcal {C}^{1}(\Omega )$ with $\Omega \subset \mathbb {R}^{2}$, see [36]. An analogous regularity result for planar AMSL extensions can be found in [34, Theorem 1].

*Main contributions:* In this work, in the framework of complete convex quasi-metric spaces with positive index of symmetry (see Section 1.1 for precise definitions), we complement the PDE perspective of the aforementioned works and provide an existence result for AMSL extensions based on an adaptation of the Perron method, namely (Theorem 2.4):

- For any semi-Lipschitz boundary data, there exists an AMSL extension.

A second contribution of the manuscript is to provide a constructive method to obtain such an AMSL extension. This is based on an (unbalanced) tug-of-war operator $u\mapsto T_r[u]$ which depends on a function $r:X\rightarrow (0,+\infty )$, see (2.11). Then, if the space is compact we construct a sequence $\{u_{\varepsilon _n}\}_n$ consisting of fixed points for adequate tug-of-war operators $T_n$ (defined through $T_r$) that converge to an AMSL extension (Theorem 2.15). This proof, in the case of a metric space is a constructive iteration scheme that guarantees the existence of an AMLE, filling the aforementioned gap in the literature.

The manuscript is organized as follows. In Section 1.1, we recall basic definitions and fix the notations. In Section 1.2 we deal with minimal semi-Lipschitz extensions, while in Section 2.1, we state our main result (existence of AMSL extensions), which will be eventually proved in Section 3.1 (Annex) via the Perron method. In Section 2.2, we present an asymmetric mean value property and establish our main convergence result, which provides an alternative proof of existence of AMSL extensions. This time, the proof is constructive, but the assumption that the space is compact is required. Finally, in Section 3.2 we provide a discussion on convex quasi-metric spaces.

### Convex quasi-metric spaces

Let us first recall the definition of a quasi-metric space.

#### Definition 1.1

(Quasi-metric space) A function $d^+: X\!\times \!X \rightarrow [0, + \infty )$ is called *quasi–metric* (or *quasi-distance*) on a nonempty set *X* if it satisfies the following conditions:

$$\mathrm {(i).}$$: $$d^+(x, y) \le d^+(x, z) + d^+(z, y)$$, for all $$x, y, z \in X$$;
$\mathrm {(ii).}$: $d^+(y, x) = d^+(x, y) = 0$ if and only if $x = y$.

In this case we say that $(X, d^+)$ (or simply *X*) is a quasi-metric space.

With respect to the classical definition of a metric space (and a distance *d*), in the above definition we do not require the property of symmetry and we allow to have $d^+(x, y) \ne d^+(y, x)$ when $x \ne y$. Notice that the function

$$ d^-(x, y) \,{:}{=}\, d^+(y, x), \quad \text {for all } x,y\in X $$

is also a quasi-metric in *X* that we call *the reverse quasi-metric* of $d^+$. Notice further that the symmetrized function

$$ d^s(x, y) \,{:}{=} \,\max \{ d^+(x, y), d^+(y, x) \} = \max \{ d^-(y, x), d^-(x, y) \} $$

is always a metric, that we call *symmetrized metric*, and the space $(X,d^s)$ is a metric space.

For a set $U \subset X$, we denote

$$\begin{aligned}&d^+(x, U) = \inf \{ d^+(x, u): u \in U \}, \\&d^-(x, U) = \inf \{ d^-(x, u): u \in U \}. \end{aligned}$$

The index of symmetry of a quasi-metric space (see [8, 38]) is defined by

1.4 $$\begin{aligned} \mathfrak {c}_X \,{:}{=}\, \inf _{d^+(x, y) > 0} \dfrac{d^+(y, x)}{d^+(x, y)}. \end{aligned}$$

We always have $\mathfrak {c}_X \in [0, 1]$. In the case $\mathfrak {c}_X > 0$, it holds

1.5 $$\begin{aligned} \mathfrak {c}_X d^+(x, y) \le d^+(y, x) \le \dfrac{1}{\mathfrak {c}_X}d^+(x, y), \quad \text { for all } x, y \in X. \end{aligned}$$

There are three natural topologies associated to a quasi–metric space $(X, d ^+)$:

$\mathrm {(i)}$: the *forward topology* $\tau ^+$, generated by the family of *forward–balls* $\lbrace B^+(x, r): x \in X, r > 0 \rbrace$, where
  $$\begin{aligned} B^+(x, r) \,{:}{=}\, \lbrace y \in X: d^+(x, y) < r \rbrace . \end{aligned}$$
$\mathrm {(ii)}$: the *backward topology* $\tau ^-$, generated by the family of *backward–balls* $\lbrace B^-(x, r): x \in X, r > 0 \rbrace$, where
  $$\begin{aligned} B^-(x, r) \,{:}{=}\, \lbrace y \in X: d^-(x, y) < r \rbrace . \end{aligned}$$
$\mathrm {(iii)}$: the *symmetric topology* $\tau ^s$, which is the topology induced by the metric $d^s$.

Notice that if the index of symmetry is positive, then thanks to (1.4) the three topologies coincide (see [8]), namely:

$$\mathfrak {c}_X > 0 \quad \implies \quad \tau ^+ = \tau ^- = \tau ^s.$$

- In this work, all topological notions (open set, closed set, boundary etc) will refer to the symmetrized topology $\tau ^s$. In particular, for any $W \subset X$, we denote by $\overline{W}$ and $\partial W$ $\tau ^s$-closure and the $\tau ^s$-boundary of *W* respectively.

In a similar spirit, we introduce the following definition.

#### Definition 1.2

(complete quasi-metric space) We say that a quasi–metric space $(X, d^+)$ is *complete*, if the corresponding metric space with the symmetrized distance $(X,d^s)$ is complete.

The reader should be alerted that sometimes the term *bicomplete* is employed to describe the notion introduced in Definition 1.2. Notice that according to our definition, the quasi-metric space $(X,d^+)$ is complete if and only if $(X,d^-)$ is complete.

The following definition is analogous to the classical metric case.

#### Definition 1.3

(convex quasi-metric space) A quasi–metric space $(X, d^+)$ is called *convex* if for every $x, y \in X$ and $r < d^+(x, y)$, there exists $z \in X$ such that

$$\begin{aligned} d^+(x, z) = r \quad \text { and } \quad d^+(z, y) = d^+(x, y) - r. \end{aligned}$$

If $(X,d^{+})$ is a *convex* space, then for every $x,y\in X$ we define the oriented segment

$$ [x,y]\,{:}{=}\,\{z\in X:\;d^{+}(x,y)=d^{+}(x,z)+d^{+}(z,y)\}. $$

Let us draw reader’s attention to the fact that the set [*x*, *y*] is not a usual segment (neither a path in the usual sense of the word) and should rather be regarded as a generalized segment, see Section 3.2 for details. Section 3.2 also contains a discussion about convex quasi–metric spaces: In particular, it is shown that $(X,d^+)$ is convex if and only if $(X,d^-)$ is convex (Lemma 3.7). However, in strong contrast to the above notion of completeness (*c.f.* Definition 1.2), there is no relation between a quasi-metric space being convex and its corresponding symmetrized metric space being convex (see Example 3.8 and Example 3.9).

### Minimal semi–Lipschitz extensions.

The notion of semi-Lipschitz function is fundamental in the study of quasi-metric spaces.

#### Definition 1.4

(semi-Lipschitz function) Let $(X, d^+)$ be a quasi-metric space. A function $f: X \rightarrow \mathbb {R}$ is called semi-Lipschitz if there exists a constant $L > 0$ such that

1.6 $$\begin{aligned} f(x) - f(y) \le L d^+(y, x), \quad \text { for all } x, y \in X. \end{aligned}$$

We denote by $\textrm{SLip}(X)$ the set of all real-valued semi-Lipschitz functions defined on *X*. For every $f\in \textrm{SLip}(X)$ we define its semi-Lipschitz constant by

$$\begin{aligned} \textrm{SLip}(f, X) \,{:}{=}\, \inf \left\{ L> 0 : L \text { satisfies~(1.6)} \right\} \, =\, \sup _{d^+(y, x) > 0}\left\{ \dfrac{\max \lbrace f(x) - f(y), 0 \rbrace }{d^+(y, x)}:\,x,y \in X \right\} . \end{aligned}$$

We also define the semi-Lipschitz constant $\textrm{SLip}(f, U)$ with respect to any nonempty subset $U\subset X$ by taking $x,y \in U$ in (1.6) and in the above supremum.

The $L^\infty$ norm of $f: X \rightarrow \mathbb {R}$ is defined by

$$\begin{aligned} \Vert f\Vert _{L^\infty (X)} = \sup _{x \in X} |f(x)|. \end{aligned}$$

#### Remark 1.5

(Continuity of semi-Lipschitz functions) Every semi-Lipschitz function *f* is $\tau ^+$–upper semicontinuous. Moreover, *f* is Lipschitz for the symmetrized metric $d^s$ and thus $\tau ^s$–continuous.

Before we proceed, let us make a comment concerning terminology. One can easily notice that in (1.6) the variables *x* and *y* do not appear with the same order at the left- and the right-hand side. The current choice ensures that the functions $d(x_0,\cdot )$ (which characterize forward convergence, in the sense that $x_n \! \xrightarrow {n \rightarrow \infty } x_0$ in the forward topology if and only if $d(x_0,x_n)\!\rightarrow 0$) are semi-Lipschitz. For an additional (deeper) reason of this choice we refer the reader to [16, Remark 2.31].

We are now consider the problem of semi-Lipschitz extensions.

#### Definition 1.6

(minimal semi-Lipschitz extension) Let *A* be a nonempty subset of *X* and ${f: A \rightarrow \mathbb {R}}$ be a semi-Lipschitz function. A function ${F: X \rightarrow \mathbb {R}}$ is called a *minimal semi-Lipschitz extension* of *f* if

$$ F|_A = f\qquad \text { and }\qquad \textrm{SLip}(F, X) = \textrm{SLip}(f, A). $$

The well–known McShane–Whitney extensions provide typical instances of minimal semi–Lipschitz extensions in every quasi-metric space, see [33]. In particular, let us denote

1.7 $$\begin{aligned} \Lambda (f)(x) \,{:}{=}\, \sup _{y \in A} \left\{ f(y) - \textrm{SLip}(f, A)\, d^+(x, y) \right\} , \quad \text { for all } x \in X, \end{aligned}$$

and

1.8 $$\begin{aligned} \Psi (f)(x) \,{:}{=}\, \inf _{y \in A} \left\{ f(y) + \textrm{SLip}(f, A) \, d^+(y, x) \right\} , \quad \text { for all } x \in X. \end{aligned}$$

The functionals $\Lambda , \Psi : \textrm{SLip}(A) \rightarrow \textrm{SLip}(X)$ ($\sup$- and $\inf$-convolutions, respectively) are the two extreme minimal extensions, in the following sense:

$$\mathrm {(i).}$$: (extendability) $$\Lambda (f)|_A = \Psi (f)|_A = f$$.
$\mathrm {(ii).}$: (minimal semi–Lipschitz constant) $\textrm{SLip}(\Lambda (f), X) = \textrm{SLip}(\Psi (f), X) = \textrm{SLip}(f, A)$.
$\mathrm {(iii).}$: (extremality) Any minimal semi–Lipschitz extension *F* of *f* satisfies:
  1.9 $$\begin{aligned} \Lambda (f) \le F \le \Psi (f). \end{aligned}$$

Let us now state the following result, which will be repeatedly used throughout the manuscript.

#### Lemma 1.7

(Key lemma) Let $(X, d^+)$ be a complete convex quasi–metric space with $\mathfrak {c}_X > 0$, $K\!\subset \!X$ be a nonempty closed set and $g: X \rightarrow \mathbb {R}$ be a minimal semi–Lipschitz extension of $g |_K$. Let $\mathcal {O} \subset X\!\setminus \!K$ be a nonempty open set and $\ell : X \rightarrow \mathbb {R}$ be a minimal semi–Lipschitz extension of $g |_{\partial \mathcal {O}}$. Then, the function

$$\begin{aligned} \varphi (x) \,{:}{=}\, {\left\{ \begin{array}{ll} \ell (x)\,, &  \quad x \in \mathcal {O}, \\ g(x)\,, &  \quad x \in X\!\setminus \!\mathcal {O}, \end{array}\right. } \end{aligned}$$

is a minimal semi–Lipschitz extension of $g |_K$. Consequently, it holds

1.10 $$\begin{aligned} \Lambda ( g |_K) \le \varphi \le \Psi ( g |_K). \end{aligned}$$

#### Proof

Notice that $\varphi = g$ on *K* (since $K\!\subset \!X\!\setminus \!\mathcal {O}$) and $\varphi |_{\partial \mathcal {O}} = g|_{\partial \mathcal {O}} = \ell |_{\partial \mathcal {O}}$ (since $\ell$ is a semi–Lipschitz extension of $g |_{\partial \mathcal {O}}$). Let us prove that $\textrm{SLip}(\varphi , X) = \textrm{SLip}(g, K)$. To this end, notice that

1.11 $$\begin{aligned} \textrm{SLip}(\varphi , \mathcal {O}) =\textrm{SLip}(\ell , \mathcal {O}) \le \underbrace{\text {SLip}(\ell , X)\,=\,\text {SLip}(g,\partial \mathcal {O})}_{\ell \text { is a minimal extension of } g|_{\partial \mathcal {O}}}\,\,\le \,\,\underbrace{\text {SLip}(g, X)\,=\,\text {SLip} (g, K)}_{g\text { is a minimal extension of } g |_K}, \end{aligned}$$

and

1.12 $$\begin{aligned} \textrm{SLip}(\varphi , X\!\setminus \!\mathcal {O}) = \textrm{SLip}(g, X\!\setminus \!\mathcal {O}) \le \textrm{SLip}(g, X) = \textrm{SLip}(g, K). \end{aligned}$$

Set $L\,{:}{=}\,\textrm{SLip}(g,K)$. We infer from (1.11) and (1.12) that $\varphi (x)-\varphi (y)\le Ld^{+}(y,x),$ provided $x,y\in \mathcal {O}$ (where $\varphi \equiv \ell$) or $x,y\in X\!\setminus \!\mathcal {O}$ (where $\varphi \equiv g$). Assume now that $x\in X\!\setminus \!\mathcal {O}$ (that is, $\varphi (x) = g(x)$) and $y\in \partial \mathcal {O}$ (that is, $\varphi (y)=\ell (y)$). Since $(X,d^+)$ is a complete, convex quasi-metric space and $\mathfrak {c}_X>0$ we can apply Lemma 3.5 (Annex) to obtain $z\in [y, x]\cap \partial \mathcal {O}$. We deduce easily by continuity of the functions $\varphi ,$ *g* and $\ell$ that $\varphi (z)=\ell (z)=g(z)$. Then

$$ \varphi (x)\,-\,\varphi (y)\,\,=\,\,\underbrace{\varphi (x)-\varphi (z)}_{g(x)-g(z)} \,\,\,\,+\,\,\underbrace{\varphi (z)-\varphi (y)}_{\ell (z)-\ell (y)}\,\,\le \,\,\underbrace{L\,d^{+}(z,x)}_{\text {by } (1.12)}\,+\,\underbrace{ L\,d^{+}(y,z)}_{\text {by } (1.12)}\,\,\underbrace{\,=\,}_{z\in [y,x]}Ld^{+}(y,x). $$

The case $x\in \mathcal {O}$ and $y\in X\!\setminus \!\mathcal {O}$ can be treated analogously. Therefore, $\varphi$ is a minimal semi–Lipschitz extension of $g |_K$. The last assertion follows from (1.9).

## Absolutely minimal semi-Lipschitz functions and extensions

Let $(X, d^+)$ be a quasi–metric space and $U \subset X$ be a nonempty open set (recall that all topological notions refer to the symmetric topology $\tau ^s$).

### Definition 2.1

(AMSL function) A function $u: U \rightarrow \mathbb {R}$ is said to be *absolutely minimal semi–Lipschitz* if it satisfies

2.1 $$\begin{aligned} \textrm{SLip}(u, V) = \textrm{SLip}(u, \partial V), \quad \text { for all } V \in \mathcal {P}(U), \end{aligned}$$

where

$$\begin{aligned} \mathcal {P}(U) \,{:}{=}\, \left\{ V \subset U : \, V \text { is open and } \overline{V} \subset U \right\} . \end{aligned}$$

In this case, we denote $u \in \textrm{AMSL}(U)$.

### Remark 2.2

(AMSL vs connectedness) A straighforward consequence of (2.1) is that $\partial V\ne \emptyset$ for every nonempty open subset *V*, that is, *X* is connected (for the $\tau ^s$-topology).

We shall now define the notion of AMSL-extension for a given boundary condition.

### Definition 2.3

(AMSL extension) Let $A \subset X$ be a nonempty closed set and $f \in \textrm{SLip}(A)$. We say that *u* is an AMSL extension of *f* if

$\mathrm {(i)}$: *u* is a minimal semi–Lipschitz extension of *f*;
$\mathrm {(ii)}$: *u* is AMSL in $X\!\setminus \!A$.

### Existence of AMSL extensions

The following theorem establishes existence of AMSL extensions for $\tau ^s$-connected convex quasi-metric spaces by using Perron method. The proof is long but follows the same scheme as in the classical case. The details of the proof are postponed and will be given in Appendix 3.1.

#### Theorem 2.4

(Existence of AMSL extension) Let $(X, d^+)$ be a complete convex quasi–metric space with $\mathfrak {c}_X > 0$. Let further *A* be a nonempty closed subset of *X* and $f \in \textrm{SLip}(A)$. Then, there exists an AMSL extension *u* of *f*.

#### Remark 2.5

(existence of boundaries) Every convex space is connected (therefore, *X* is $\tau ^{+}$-connected). In Theorem 2.4 we implicitly assume that *X* is connected with respect to the symmetric topology (since $\mathfrak {c}_X>0$ yields $\tau ^{s}=\tau ^{+}$), which ensures that the boundary $\partial V$ of every nonempty open set *V* is nonempty (*c.f.* Remark 2.2).

To end this section, we discuss a slightly different notion of AMLEs proposed by Juutinen in [21]. In that work, the author characterized AMLEs in an alternative manner: Let *X* be a metric space, $A \subset X$, $f \in \text {Lip}(A)$ and $u: X \rightarrow \mathbb {R}$ be a Lipschitz function. A function *u* is AMLEs (associated with boundary data *f*) if it satisfies:

$\mathrm {(i).}$: *u* is a minimal Lipschitz extension of *f*;
$\mathrm {(ii).}$: for every $V \subset X$ and $\varphi : X \rightarrow \mathbb {R}$ minimal Lipschitz extension of *f* such that $\varphi = u$ in $X\!\setminus \!V$, one has $\text {Lip}(u, V) \le \text {Lip}(\varphi , V)$.

An advantage of this definition is that it avoids the appearance of the boundary in the notion of AMLE. In Proposition 2.6, using the fact that every semi–Lipschitz function is continuous (*c.f.* Remark 1.5) we show that AMSL extensions, associated to ${\mathcal {P}(X\!\setminus \!A)}$, can also be viewed from Juutinen’s perspective.

#### Proposition 2.6

Let *X* be a complete convex quasi-metric space with $\mathfrak {c}_X>0$. Let further $A\subset X$ be nonempty closed and $f\in \text {SLip}(A)$. The following assertions are equivalent.

$\mathrm {(i).}$: *u* is an *AMSL* extension of *f*.
$\mathrm {(ii).}$: *u* is a minimal semi–Lipschitz extension of *f* and for every $V \in \mathcal {P}(X\!\setminus \!A)$ and $\varphi \in \textrm{SLip}(X)$ minimal semi–Lipschitz extension of *f* such that $\varphi = u$ on $X\!\setminus \!V$, it holds
  2.2 $$\begin{aligned} \textrm{SLip}(u, V) \le \textrm{SLip}(\varphi , V). \end{aligned}$$

#### Proof

$\mathrm {(i)}\Rightarrow \mathrm {(ii)}$. Let *u* be an *AMSL* extension of *f*. Fix $V\in \mathcal {P}(X\!\setminus \!A)$ and a minimal semi–Lipschitz extension $\varphi$ of *f* such that $\varphi =u$ in $X\!\setminus \!V$. Since $\partial V \subset X\!\setminus \!V$, we have $\varphi |_{\partial V}=u|_{\partial V}$. In addition, continuity of $\varphi$ (*c.f.* Remark 1.5) yields that $\text {SLip}(\varphi ,V)=\text {SLip}(\varphi ,\overline{V})$. Since $u\in \text {AMSL}(X\!\setminus \!A)$, we obtain

$$ \text {SLip}(u,V)\underbrace{=}_{u\text { is AMSL}}\text {SLip}(u,\partial V)\underbrace{=}_{u|_{\partial V}=\varphi |_{\partial V}}\text {SLip} (\varphi ,\partial V)\le \text {SLip}(\varphi ,\overline{V})=\text {SLip} (\varphi ,V). $$

$\mathrm {(ii)}\Rightarrow \mathrm {(i)}$. Let us assume that $u\in \text {SLip}(X)$ satisfies $\mathrm {(ii)}$. Then for any $V\in \mathcal {P}(X\!\setminus \!A)$, we set

$$ \varphi (x)=\left\{ \begin{array}{ll} \Psi (u|_{\partial V})(x), &  \quad x\in V\\ \quad u(x), &  \quad x\in X\!\setminus \!V \end{array} \right. . $$

Notice that $\varphi =u=f$ on *A* (since $A\subset X\!\setminus \!V$) and $\varphi |_{\partial V}=u|_{\partial V}$. Applying Lemma 1.7 to the case $K = A$, $g = u$, $\mathcal {O} = V$ and $\ell = \Psi ( u |_{\partial V})$, we infer that $\varphi$ is a minimal extension of $u |_K \equiv f$. Therefore, $\varphi$ can be used as a test function to deduce:

$$ \text {SLip}(u,V)\underbrace{\le }_{(2.2)}\text {SLip} (\varphi ,V)=\text {SLip}(\Psi (u|_{\partial V}),V)\le \text {SLip}(\Psi (u|_{\partial V}),X)=\text {SLip}(u,\partial V). $$

We deduce that $u\in \text {AMSL}(X\!\setminus \!A)$, which completes the proof.

### Obtaining AMSL extensions via a constructive scheme

Borrowing ideas from the work of Le Gruyer and Archer [23, 24], we present an approximation scheme for AMSL extensions on compact quasi–metric spaces. Concurrently, this approximation provides an alternative proof for the existence of AMSL extensions (Theorem 2.4), beyond the Perron method.

#### A preliminary technical lemma

We are going to define a tug-of-war operator, analogous to (1.3) (considered in [23, 24]) which is paramount for our purposes. However, there will be two fundamental differences between the latter operator and the one we introduce hereby (see forthcoming definition (2.11)), namely:

(i) a flexibility on the radius of the balls, that we now allow to vary with the given point; and
(ii) a combined use of forward and backward balls (leading to an unbalanced tug-of-war operator).

Notice that (i) will also provide a constructive method for obtaining AMLE in case of metric spaces (see discussion in the introduction). In the case of metric spaces there is no difference between forward and backward balls and (ii) is irrelevant.

In order to formalize (i), let us now consider any (continuous) function $r:X\rightarrow [0,+\infty )$ which is 1-semi-Lipschitz for both the forward and the backward distance, that is,

2.3 $$\begin{aligned} r(x)-r(y)\le \min \{d^{+}(x,y),d^{+}(y,x)\},\quad \text { for all }x,y\in X. \end{aligned}$$

We shall first need the following technical lemma, in which the above function will be used to determine the radius of forward (respectively, backward) balls at every $x\in X.$

##### Lemma 2.7

Let $(X,d^{+})$ be a convex quasi-metric space and let $r:X\rightarrow [0,+\infty )$ satisfy (2.3). Then for every $x,y\in X$ we have

2.4 $$\begin{aligned} \sup _{a\in \overline{B^{+}(x,r(x))}}\,\,\,\inf _{b\in \overline{B^{+}(y,r(y))}} d^{+}(b,a)\,\,\le \,\, d^{+}(y,x)+r(x)-r(y) \end{aligned}$$

and

2.5 $$\begin{aligned} \sup _{b^{\prime }\in \overline{B^{-}(y,r(y))}}\,\,\inf _{a^{\prime }\in \overline{B^{-}(x,r(x))}}d^{+}(b^{\prime },a^{\prime })\,\,\le \,\,d^{+} (y,x)+r(y)-r(x) \end{aligned}$$

##### Proof

Let us establish (2.4). To this end, observe that

2.6 $$\begin{aligned} \sup _{a\in \overline{B^{+}(x,r(x))}\cap \overline{B^{+}(y,r(y))}}\,\,\inf _{b\in \overline{B^{+}(y,r(y))}}d^{+}(b,a)\,=\,0\underbrace{\le }_{\text {(2.3)}}d^{+}(y,x)+r(x)-r(y). \end{aligned}$$

Let now $a\in \overline{B^{+}(x,r(x))}\diagdown \overline{B^{+}(y,r(y))}.$ Then $d^{+}(y,a)>r(y).$ Since $(X,d^{+})$ is a convex quasi-metric space, there exists $z\in X$ such that

$$ d^{+}(y,z)=r(y)\qquad \text {and}\qquad d^{+}(z,a)=d^{+}(y,a)-r(y). $$

It follows that

2.7 $$\begin{aligned} \inf _{b\in \overline{B^{+}(y,r(y))}}d^{+}(b,a)\le d^{+}(z,a)=d^{+} (y,a)-r(y). \end{aligned}$$

On the other hand, for every $b\in \overline{B^{+}(y,r(y))}$ by triangle inequality we deduce:

$$ d^{+}(y,a)\le d^{+}(y,b)+d^{+}(b,a)\le r(y)+d^{+}(b,a), $$

yielding

$$ d^{+}(y,a)-r(y)\le d^{+}(b,a) $$

and consequently,

2.8 $$\begin{aligned} d^{+}(y,a)-r(y)\le \inf _{b\in \overline{B^{+}(y,r(y))}}d^{+}(b,a). \end{aligned}$$

Combining (2.7) and (2.8) we obtain

2.9 $$\begin{aligned} \inf _{b\in \overline{B^{+}(y,r(y))}}d^{+}(b,a)=d^{+}(y,a)-r(y). \end{aligned}$$

It follows directly that

$$ {\begin{matrix} & \sup _{a\in \overline{B^{+}(x,r(x))}\setminus \overline{B^{+}(y,r(y))}}\left( \inf _{b\in \overline{B^{+}(y,r(y))}}d^{+}(b,a)\right) \\ & \qquad \le ~\sup _{a\in \overline{B^{+}(x,r(x))}\setminus \overline{B^{+}(y,r(y))}} d^{+}(y,a)-r(y)\\ & \qquad \le ~ d^{+}(y,x)+\sup _{a\in \overline{B^{+}(x,r(x))}\setminus \overline{B^{+}(y,r(y))}}d^{+}(x,a)-r(y)\\ & \qquad \le ~ d^{+}(y,x)+r(x)-r(y). \end{matrix}} $$

Taking into account (2.6), inequality (2.4) follows. Inequality (2.5) is obtained in an analogous way.

We immediately obtain the following corollary:

##### Corollary 2.8

Let $(X,d^{+})$ be a quasi-metric space. Then for any function $r:X\rightarrow (0,+\infty )$ satisfying (2.3) and any $x,y\in X$ it holds:

2.10 $$\begin{aligned} \sup _{a\in \overline{B^{+}(x,r(x))}}\,\,\,\inf _{b\in \overline{B^{+}(y,r(y))}} d^{+}(b,a)\,+\,\sup _{b^{\prime }\in \overline{B^{-}(y,r(y))}}\,\,\inf _{a^{\prime }\in \overline{B^{-}(x,r(x))}}d^{+}(b^{\prime },a^{\prime })\,\le \,2d^{+}(y,x). \end{aligned}$$

#### Unbalanced harmonious operator and comparison principle

We now introduce the main tool for our construction. (The terminology *harmonious* was first employed in [24] and refers to the averaging of the maximal and the minimal value over a ball, in contrast to *harmonic* that would average over all values.)

##### Definition 2.9

(unbalanced harmonious regularization operator) Given $r:X\rightarrow [0,+\infty )$ the unbalanced harmonious regularization operator $T_r: \textrm{SLip}(X) \rightarrow \textrm{SLip}(X)$ is defined for every $u \in \textrm{SLip}(X)$ as follows:

2.11 $$\begin{aligned} T_r[u](x) \,{:}{=}\, \dfrac{1}{2} \left( \sup _{a \in \overline{B^+(x, r(x))} } u(a) + \inf _{b \in \overline{B^-(x, r(x))} } u(b) \right) , \, \text { for all } x \in X. \end{aligned}$$

Notice that for every $\bar{x}\in X$ for which $r(\bar{x})=0$, the above formula yields $T_r[u](\bar{x})=u(\bar{x})$.

##### Remark 2.10

(i). If *X* is a metric space, then $B^+(x, \varepsilon ) = B^-(x, \varepsilon )$ for all $x \in X$ and $\varepsilon > 0$. Taking $r(x)=\min \{ \varepsilon , \text {dist}(x, A) \}$ for all $x\in X$ the operator (2.11) becomes the one used in [24].
(ii). After straightforward calculations we deduce from Corollary 2.8 and the above definition of $T_r$, that
  $$\begin{aligned} \textrm{SLip}(T_r[u], X) \le \textrm{SLip}(u, X),\quad \text { for all } u \in \textrm{SLip}(X). \end{aligned}$$
(iii). It is easy to see that for every $u, v \in \textrm{SLip}(X)$ we have
  $$\begin{aligned} \left\| T_r[u] - T_r[v] \Vert _{L^{\infty }(X)} \le ~ \right\| u - v \Vert _{L^{\infty }(X)}. \end{aligned}$$

In what follows, we fix a nonempty closed subset *A* of *X* and consider the function:

2.12 $$\begin{aligned} h(x)\,{:}{=}\,\min \{d^{+}(x,A),d^{-}(x,A)\},\quad \text { for all }x\in X. \end{aligned}$$

##### Lemma 2.11

(Comparison Principle for $T_r$) Let $(X, d^+)$ be a compact convex quasi–metric space with $\mathfrak {c}_X > 0$, $A\subset X$ a nonempty closed set and $f \in \textrm{SLip}(A)$. Let us further assume that $r: X \rightarrow [0,+\infty )$ satisfy (2.3) and be such that:

2.13 $$\begin{aligned} r(x) = 0\quad \iff \quad x \in A. \end{aligned}$$

Let $u \in \textrm{SLip}(X)$ and $v \in \textrm{SLip}(X)$ respectively satisfy

2.14 $$\begin{aligned} {\left\{ \begin{array}{ll} u(x) \le T_r[u](x), &  \, x \in X\!\setminus \!A, \\ u(x) \le f(x), &  \, x \in A, \end{array}\right. } \end{aligned}$$

and

2.15 $$\begin{aligned} {\left\{ \begin{array}{ll} v(x) \ge T_r[v](x), &  \, x \in X\!\setminus \!A, \\ v(x) \ge f(x), &  \, x \in A. \end{array}\right. } \end{aligned}$$

Then:

$$\begin{aligned} \Delta \,{:}{=}\,\max _{x \in X} (u - v)(x) \le 0. \end{aligned}$$

##### Proof

For $\Delta \,{:}{=}\,\max _{x\in X}(u-v)(x)$ we set

$$\begin{aligned}&\mathcal {W}\,{:}{=}\,\underset{X}{\textrm{argmax}}\,(u-v)\,\equiv \left\{ y\in X:(u-v)(y)=\Delta \right\} , \\&\widehat{\mathcal {W}}\,{:}{=}\,\underset{\mathcal {W}}{\textrm{argmax}} \,u\,\equiv \left\{ z\in \mathcal {W}:u(z)=M\right\} \quad \left( \text { where }M\,{:}{=}\,\max _{x\in \mathcal {W}}u(x)\right) . \end{aligned}$$

Notice that if $\bar{a}\in A\cap \mathcal {W}$ then

$$ u(\bar{a})\underbrace{\le }_{(2.14)}f(\bar{a})\underbrace{\le }_{(2.15)}v(\bar{a})\qquad \text {yielding }\quad \Delta \,{:}{=}\,(u-v)(\bar{a})\le 0 $$

and the conclusion follows. Consequently, it suffices to establish that $A\cap \mathcal {W}\ne \emptyset$.

Let us assume, towards a contradiction, that $A\cap \mathcal {W} =\emptyset ,$ or equivalently, $\mathcal {W}\subset X\setminus A$.

Recalling (2.12), since *A* is closed and $\widehat{\mathcal {W}}$ is compact, there exists $\bar{w}\in \widehat{\mathcal {W}}$ such that

2.16 $$\begin{aligned} h(\bar{w})=\inf _{w\in \widehat{\mathcal {W}}}h(w)\,{:}{=}\,d_{0}>0. \end{aligned}$$

Furthermore, without the loss of generality, we may assume that

$$ h(\bar{w})=d^{+}(\bar{w},A)=d^{+}(\bar{w},\bar{a}),\quad \text {for some } \bar{a}\in A. $$

Since $r(\bar{a})=0$, it follows from (2.3) that $0<r(\bar{w})\le d^{+}(\bar{w},\bar{a} )$.

*Claim.* For every $x\in \widehat{\mathcal {W}}$ we have

2.17 $$\begin{aligned} \overline{B^{+}(x,r(x))}\cap \overline{B^{-}(x,r(x))}\subset \widehat{\mathcal {W}}\text {.} \end{aligned}$$

##### Proof of the Claim

Assume that for some $\bar{x} \in \widehat{\mathcal {W}}$ we have $\overline{B^{+}(\bar{x},r(\bar{x}))} \cap \overline{B^{-}(\bar{x},r(\bar{x}))}\not \subset \widehat{\mathcal {W}}$. Since *X* is compact, so are its closed balls. Therefore, there exists $\bar{z}\in \overline{B^{+}(\bar{x} ,r(\bar{x}))}$ such that

$$ u(\bar{z})=\max _{y\in \overline{B^{+}(\bar{x},r(\bar{x}))}}u(y)\ge u(\bar{x})=M. $$

Since $\bar{x}\in \widehat{\mathcal {W}}\subset \mathcal {W}\subset X\diagdown A$ we deduce from (2.14) and (2.15) that

$$\begin{aligned} \Delta =&~(u-v)(\bar{x})~\le ~T_{r}[u](\bar{x})-T_{r}[v](\bar{x})\\ =&~\dfrac{1}{2}\left( \max _{y\in \overline{B^{+}(\bar{x},r(\bar{x}))} }u(y)-\max _{y\in \overline{B^{+}(\bar{x},r(\bar{x}))}}v(y)\right) +\dfrac{1}{2}\left( \min _{y\in \overline{B^{-}(\bar{x},r(\bar{x}))}}u(y)-\min _{y\in \overline{B^{-}(\bar{x},r(\bar{x}))}}u(y)\right) \\ \le&~\dfrac{1}{2}\left( \max _{y\in \overline{B^{+}(\bar{x},r(\bar{x}))} }u(y)-\max _{y\in \overline{B^{+}(\bar{x},r(\bar{x}))}}v(y)\right) +\dfrac{1}{2}\Delta , \end{aligned}$$

which eventually leads to

2.18 $$\begin{aligned} \Delta =\max _{y\in \overline{B^{+}(\bar{x},r(\bar{x}))}}u(y)-\max _{y\in \overline{B^{+}(\bar{x},r(\bar{x}))}}v(y). \end{aligned}$$

Let us consider the following two cases.

*Case 1*: $u(\bar{z})>M$. By definition of $\mathcal {W}$ and *M*, we deduce that $\bar{z}\not \in \mathcal {W}$. On the other hand, thanks to the identity (2.18), we have $\Delta \le (u-v)(\bar{z})\le \Delta$, that is, $\bar{z}\in \mathcal {W}$, which is a contradiction.

*Case 2*: $u(\bar{z})=M$. Then $u(\bar{x})=u(\bar{z})=M$ and we get from (2.14) that

$$ M=u(\bar{x})\le \dfrac{1}{2}\left( \underbrace{\max _{y\in \overline{B^{+}(\bar{x} ,r(\bar{x}))}}u(y)}_{=u(\bar{z}) =M}+\min _{y\in \overline{B^{-}(\bar{x},r(\bar{x}))}}u(y)\right) =\dfrac{1}{2}\left( M+\min _{y\in \overline{B^{-}(\bar{x},r(\bar{x}))} }u(y)\right) , $$

which leads to

$$ \min _{y\in \overline{B^{-}(\bar{x},r(\bar{x}))}}u(y)\ge M. $$

It follows that

2.19 $$\begin{aligned} u(y)=M,\qquad \text { for all } \,\, y\in \overline{B^{+}(\bar{x},r(\bar{x}))} \cap \overline{B^{-}(\bar{x},r(\bar{x}))}. \end{aligned}$$

Since

$$ \overline{B^{+}(\bar{x},r(\bar{x}))}\cap \overline{B^{-}(\bar{x},r(\bar{x} ))}\not \subset \widehat{\mathcal {W}}, $$

there exists

$$ w\in \overline{B^{+}(\bar{x},r(\bar{x}))}\cap \overline{B^{-} (\bar{x},r(\bar{x}))}\setminus \widehat{\mathcal {W}}. $$

It follows from (2.19) that $w\notin \mathcal {W}$ and consequently, $(u-v)(w)<\Delta$. Using (2.18) we get

$$ \Delta =\underbrace{\max _{y\in \overline{B^{+}(\bar{x},r(\bar{x}))}}u(y)}_{=u(w)}-\underbrace{\max _{y\in \overline{B^{+}(\bar{x},r(\bar{x}))}}v(y)}_{\ge v(w)}\,\le (u-v)(w)\,<\Delta , $$

which is a contradiction. This completes the proof of the claim. $\square$

It follows from the above claim that the (closed) set $\widehat{\mathcal {W}}$ is also open. Since *X* is connected (see Lemma 3.5) and $\emptyset \ne A \subset X \setminus \widehat{\mathcal {W}}$, it follows that $\widehat{\mathcal {W}}$ must be empty, which is a contradiction. Therefore, $\mathcal {W} \cap A \ne \emptyset$, which completes the proof. $\square$

Every function *u* satisfying (2.14) is called *subsolution* of the equation

2.20 $$\begin{aligned} T_r[u]=u. \end{aligned}$$

In an analogous way, every function *v* satisfying (2.15) is called *supersolution* of (2.20). Before we proceed further, let us notice that the constant functions

$$ \underline{u} \equiv \min _{a \in A} f(a)\qquad \text { and }\qquad \overline{v} \equiv \max _{a \in A} f(a) $$

are respectively subsolution and supersolution of (2.20).

As a direct consequence of Lemma 2.11 and the above remark, we obtain the following *maximum principle* associated with the operator $T_r$.

##### Corollary 2.12

(Maximum principle) In the framework of Lemma 2.11, the following assertions hold true.

$(\textrm{i})$.: If $u \in \textrm{SLip}(X)$ satisfies (2.14), then one has $\underset{x\in X}{\max }\, u(x) \,\le \,\overline{v} \equiv \underset{a\in A}{\max } f(a)$.
$(\textrm{ii})$.: If $v \in \textrm{SLip}(X)$ satisfies (2.15), then one has $\underset{x\in X}{\min }\, v(x) \,\ge \, \underline{u} \equiv \underset{a\in A}{\min } \,f(a)$.

#### Obtaining AMSL extentions via an iteration scheme

In this part we produce an iterative scheme that converges to an AMSL extension starting from any semi-Lipschitz boundary data. We first need to establish the following fixed-point theorem for every unbalanced harmonious operator $T_r$, where $r:X\rightarrow [0,+\infty )$.

##### Theorem 2.13

(existence of fixed points for $T_r$) Let $(X, d^+)$ be a compact convex quasi-metric space with $\mathfrak {c}_X > 0$. Let further $A \subset X$ be a nonempty closed set, $f \in \textrm{SLip}(A)$ and $r: X \rightarrow [0,+\infty )$.

The following assertions hold:

$\mathrm {(i)}$: There exists a minimal semi-Lipschitz extension $u_r \in \textrm{SLip}(X)$ of *f* such that $T_r[u_r] = u_r$.
$\mathrm {(ii)}$: If *r* satisfies (2.13) then $u_r$ is unique.

##### Proof

(i). Let $\mathcal {K}$ be the set of minimal semi-Lipschitz extensions $u \in \textrm{SLip}(X)$ of *f* such that

2.21 $$\begin{aligned} \Vert u \Vert _{L^{\infty }(X)}\, \le \, \Vert f \Vert _{L^{\infty }(A)} + \textrm{SLip}(f, A)\, \text {diam}(X), \end{aligned}$$

where $\text {diam}(X)\,{:}{=}\,\sup \,\{\,d^+(x,y):\,x,y\in X \}$. Notice that the set $\mathcal {K} \subset \textrm{SLip}(X)$ is nonempty since $\Psi (f) \in \mathcal {K}$. Moreover, $\mathcal {K}$ is a closed convex subset of $C((X, d^s), \mathbb {R})$, which is the space of real-valued $\tau ^s$–continuous functions on *X*. Notice that every minimal semi-Lipschitz extension *u* of *f* is also Lipschitz continuous with respect to the symmetric distance $d^s$. Moreover,

$$ \textrm{Lip}(u, X)\le \textrm{SLip}(u, X) = \textrm{SLip}(f,A), $$

that is, all Lipschitz constants are uniformly bounded by $\textrm{SLip}(f, A)$. Therefore, we can apply Arzelà–Ascoli theorem to deduce that the set $\mathcal {K}$ is compact in $C((X, d^s), \mathbb {R})$. Moreover, thanks to Remark 2.10 (iii), the operator $T_r: \mathcal {K} \rightarrow \mathcal {K}$ is nonexpansive with respect to the $L^\infty$ norm. Using Schauder fixed point theorem, there exists $u_r \in \mathcal {K}$ and $T_r[u_r] = u_r$.

(ii). Uniqueness of $u_r$ follows directly from Lemma 2.11.

##### Remark 2.14

In the framework of Theorem 2.13 (ii), let *u* denote the unique minimal semi-Lipschitz extension of *f* such that $T_r[u] = u$. Applying Mann–Ishikawa iterations to the nonexpansive map $T_r$ (see [19, 25]), we can approximate *u* by an iterative scheme $\{ u_n \}$ as follows. Let $\{ \alpha _n \} \subset (0, 1)$ be a sequence such that $\lim _{n \rightarrow \infty } \alpha _n = 0 \text { and } \sum _{n = 1}^{\infty } \alpha _n = + \infty$. Let $u_0$ be any minimal semi-Lipschitz extension of *f*, for example, $u_0 = \Psi (f)$. For each $n \in \mathbb {N}$, we define

2.22 $$\begin{aligned} u_{n + 1}(x) = (1 - \alpha _n) u_n(x) + \alpha _n T_r[u_n](x), \quad \text { for all } x \in X. \end{aligned}$$

As shown in [13, Theorem 1], the convergence rate of this iterative scheme is

2.23 $$\begin{aligned} \left\| u_n - T_r[u_n] \right\| _{L^\infty (X)} \le \dfrac{2\bar{C}}{\sqrt{\pi \sum _{i = 1}^n \alpha _i (1 - \alpha _i)}}, \text { where } \bar{C} = \Vert f \Vert _{L^\infty (A)} \,+ \,\textrm{SLip}(f, A) \,\text {diam}(X). \end{aligned}$$

**Fig. 1:**
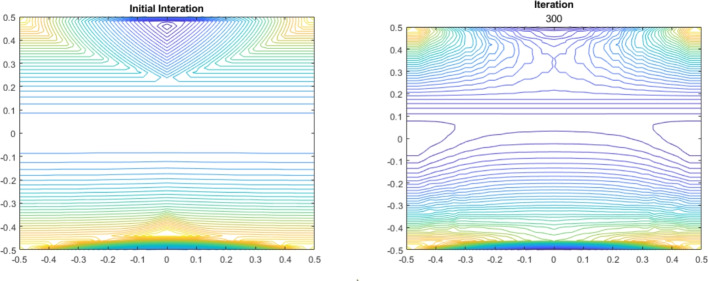
The Mann–Ishikawa algorithm is considered with $\alpha _n = \log _{10}(k^{10} + e^{12})$ and executed in MATLAB R2023a. The plots show the contour lines of the initial iteration $u_0 = (1/2)(\Lambda (f) + \Psi (f))$ and the 300*th* iteration. Error at 300*th* iteration computed via $\Vert u_n - T_r[u_n] \Vert _{L^\infty (X)}$ is 0.0607.

Figure 1 shows the Mann–Ishikawa iterations for the following example:

Let $$X = [-0.5, 0.5] \times [-0.5, 0.5]$$ and

$$ d^+((x_1, x_2), (y_1, y_2)) = \sqrt{(x_1 - y_1)^2 + 4\max \{0, x_2 - y_2 \}^2 + 100 \max \{0, y_2 - x_2 \}^2}, $$

for all $(x_1, x_2), (y_1, y_2) \in X$. It is straightforward to see that $\mathfrak {c}_X > 0$ and $(X, d^+)$ is compact. We consider $A = ([-0.5, 0.5] \times \{ - 0.5, 0.5 \}) \cup (\{ - 0.5, 0.5 \} \times [-0.5, 0.5])$ and $f: A \rightarrow \mathbb {R}$ defined by $f(x_1, x_2) = \sin (x_1^2) + x_2^2$.

We now illustrate how this constructive scheme, based on the fixed point result of Theorem 2.13, ensures the existence of an AMSL extension. To this end, for every $\varepsilon > 0$, let us denote

2.24 $$\begin{aligned} r_{\varepsilon }(x) \,{:}{=}\, \mathfrak {c}_X \min \lbrace \varepsilon , h(x) \rbrace , \quad \text { for every } x \in X, \end{aligned}$$

where the function *h* is given by (2.12). Then, Theorem 2.13 guarantees that there exists a minimal semi–Lipschitz extension $u_{\varepsilon }: X \rightarrow \mathbb {R}$ of *f* such that $u_\varepsilon = T_{r_\varepsilon }[u_\varepsilon ]$. Notice that

$$\begin{aligned} \textrm{Lip}(u_\varepsilon , X) \le \textrm{SLip}(f, A). \end{aligned}$$

Therefore, by Arzelà–Ascoli theorem, there exists a sequence $\{ \varepsilon _n \}_n$ and a function $\bar{u} \in \textrm{SLip}(X)$ such that $\lim _{n \rightarrow \infty } \varepsilon _n = 0$,

2.25 $$\begin{aligned} u_{\varepsilon _n} \rightarrow \bar{u} \quad \text { (uniformly as }n \rightarrow \infty ) \end{aligned}$$

It follows that $\bar{u}$ is a minimal semi–Lipschitz extension of *f*. We shall now show that $\bar{u}$ is in fact an absolutely minimal semi-Lipschitz extension of *f*.

##### Theorem 2.15

(constructing an AMSL extension) In the framework of Theorem 2.13, let $\bar{u}$ be given by (2.25) above. Then there exists a constant $C > 0$ such that for each $\delta > 0$ and $V \in \mathcal {P}(X\!\setminus \!A)$, there exists $N \in \mathbb {N}$ such that for all $n \ge N$, it holds

2.26 $$\begin{aligned} \bar{u}(x)\, \le \, \min _{y \in \partial V} \{ \bar{u}(y) + \textrm{SLip}(\bar{u}, \partial V) d^+(y, x) \} + 2\delta + C \varepsilon _n, \quad \text { for all } x \in \overline{V}, \end{aligned}$$

and

2.27 $$\begin{aligned} \bar{u}(x) \, \ge \, \max _{y \in \partial V} \{ \bar{u}(y) - \textrm{SLip}(\bar{u}, \partial V) d^+(x, y) \} - 2\delta - C \varepsilon _n, \quad \text { for all } x \in \overline{V}. \end{aligned}$$

In particular, $\bar{u}$ is an AMSL extension of *f*.

##### Proof

Let us prove (2.26). Fix $\delta > 0$ and $V \in \mathcal {P}(X\!\setminus \!A)$. Recalling (2.12), we notice that $h(x)>0$, for all $x\in \overline{V}$. Let $N \in \mathbb {N}$ be sufficiently large such that for every $n \ge N$ one has

2.28 $$\begin{aligned} \Vert u_{\varepsilon _n} - \bar{u} \Vert _{L^\infty (X)} \le \delta \qquad \text { and } \qquad h(x) > \varepsilon _n, \text { for all } x \in \overline{V}. \end{aligned}$$

Fix $n \ge N$. From (2.24) we get that $r_{\varepsilon _n}(x) = \mathfrak {c}_X \varepsilon _n$ for all $x \in V$. Define

$$\begin{aligned} \widehat{r}_{\varepsilon _n}(x) = \mathfrak {c}_X \min \left\{ \varepsilon _n, \widehat{h}(x) \right\} , \quad \text { for all } x \in \overline{V}, \end{aligned}$$

where

$$\begin{aligned} \widehat{h}(x) = \min \{ d^+(x, \partial V), d^-(x, \partial V) \}, \quad \text { for all } x \in \overline{V}. \end{aligned}$$

Applying Theorem 2.13 for $X=\overline{V}$, $A=\partial V$ and $f=\bar{u}|_{\partial V}$, we obtain a minimal semi–Lipschitz extension $v_{\varepsilon _n}: \overline{V} \rightarrow \mathbb {R}$ of $\bar{u} \vert _{\partial V}$ such that

$$\begin{aligned} v_{\varepsilon _n}(x) = T_{\widehat{r}_{\varepsilon _n}}[v_{\varepsilon _n}](x), \quad \text { for all } x \in \overline{V}. \end{aligned}$$

Set

$$\begin{aligned}&\mathcal {W} \,{:}{=}\, \left\{ x \in \overline{V}: \, (u_{\varepsilon _n} - v_{\varepsilon _n})(x) = \max _{y \in \overline{V}} \, (u_{\varepsilon _n} - v_{\varepsilon _n})(y) \right\} , \\&\widehat{\mathcal {W}} \,{:}{=}\, \left\{ x \in \mathcal {W}: \, u_{\varepsilon _n}(x) = \max _{y \in \mathcal {W}} u_{\varepsilon _n}(y) \right\} . \end{aligned}$$

Note that $\mathcal {W}$ and $\widehat{\mathcal {W}}$ are nonempty since *X* is compact. Let $\bar{x} \in \widehat{\mathcal {W}}$ be such that

$$\begin{aligned} \widehat{h}(\bar{x}) = \min _{y \in \widehat{\mathcal {W}}} \widehat{h}(y). \end{aligned}$$

*Claim.* $$\widehat{h}(\bar{x}) \le \varepsilon _n$$.

##### Proof of Claim

Reasoning towards a contradiction, let us assume that $\widehat{h}(\bar{x}) > \varepsilon _n$. Consequently, we have $\widehat{r}_{\varepsilon _n}(\bar{x}) = r_{\varepsilon _n}(\bar{x}) = \mathfrak {c}_X \varepsilon _n$. Therefore,

$$ B^+(\bar{x}, \widehat{r}_{\varepsilon _n}(\bar{x})) = B^+(\bar{x}, r_{\varepsilon _n}(\bar{x})) = B^+(\bar{x}, \mathfrak {c}_X \varepsilon _n), $$

$$ B^-(\bar{x}, \widehat{r}_{\varepsilon _n}(\bar{x})) = B^-(\bar{x}, r_{\varepsilon _n}(\bar{x})) = B^-(\bar{x}, \mathfrak {c}_X \varepsilon _n), $$

and

$$ B^+(\bar{x}, \mathfrak {c}_X \varepsilon _n) \cap B^-(\bar{x}, \mathfrak {c}_X \varepsilon _n) \subset V. $$

Without the loss generality, we assume that $\widehat{h}(\bar{x}) = d^+(\bar{x}, \partial V) = d^+(\bar{x}, \bar{w})$ for some $\bar{w} \in \partial V$. Take $\varrho \in (0, \mathfrak {c}_X \varepsilon )$ sufficiently small to ensure

$$ \overline{B^+(\bar{x}, \varrho )} \subset B^+(\bar{x}, \mathfrak {c}_X \varepsilon _n) \cap B^-(\bar{x}, \mathfrak {c}_X \varepsilon _n) $$

Since $(X, d^+)$ is convex, there exists $\bar{z} \in X$ such that

$$\begin{aligned} d^+(\bar{x}, \bar{z}) = \varrho \quad \text { and } \quad d^+(\bar{z}, \bar{w}) = d^+(\bar{x}, \bar{w}) - \varrho . \end{aligned}$$

Therefore, $\bar{z} \in \overline{B^+(\bar{x}, \varrho )}$. With analogous arguments used in the proof of Lemma 2.11, we infer that $B^+(\bar{x}, \mathfrak {c}_X \varepsilon _n) \cap B^-(\bar{x}, \mathfrak {c}_X \varepsilon _n) \subset \widehat{\mathcal {W}}$. As a consequence, we obtain

$$ \widehat{h}(\bar{z}) \le d^+(\bar{z}, \bar{w}) < d^+(\bar{x}, \bar{w}) = \min _{y \in \widehat{\mathcal {W}}} \widehat{h}(y), $$

which is a contradiction and the claim is proved. $\square$

Let us now prove (2.26). Since $v_{\varepsilon _n}$ is a minimal semi–Lipschitz extension of $\bar{u} \vert _{\partial V}$, we know that

2.29 $$\begin{aligned} v_{\varepsilon _n}(x) \le \underbrace{\min _{y \in \partial V} \{ \bar{u}(y) + \textrm{SLip}(\bar{u}, \partial V) d^+(y, x) \}}_{\Psi (\bar{u}|_{\partial V})}, \quad \text { for all } x \in \overline{V}. \end{aligned}$$

Using the estimate (2.28) and the fact that $\bar{x} \in \widehat{\mathcal {W}}$, we have for each $x \in V$

2.30 $$\begin{aligned} (\bar{u} - v_{\varepsilon _n})(x) = (\bar{u} - u_{\varepsilon _n})(x) + (u_{\varepsilon _n} - v_{\varepsilon _n})(x) \le \delta + (u_{\varepsilon _n} - v_{\varepsilon _n})(\bar{x}). \end{aligned}$$

Assuming (without the loss of generality) that $\widehat{h}(\bar{x}) = d^+(\bar{x}, \bar{y})$ for some $\bar{y} \in \partial V \subset \overline{V}$ and using the fact that $v_{\varepsilon _n} = \bar{u}$ on $\partial V$ and $h(\bar{x}) \le \varepsilon _n$ we deduce:

2.31 $$\begin{aligned} {\begin{matrix} (u_{\varepsilon _n} - v_{\varepsilon _n})(\bar{x}) = &  ~ u_{\varepsilon _n}(\bar{x}) - u_{\varepsilon _n}(\bar{y}) + u_{\varepsilon _n}(\bar{y}) - \bar{u}(\bar{y}) + \overbrace{v_{\varepsilon _n}(\bar{y})}^{\bar{u}(\bar{y})} - v_{\varepsilon _n}(\bar{x}) \\ \le \, &  ~ \textrm{SLip}(f, A) \underbrace{d^+(\bar{y}, \bar{x})}_{h(\bar{x})\le \varepsilon _n} + \delta + \textrm{SLip}(f, A) \underbrace{d^+(\bar{x}, \bar{y})}_{\le \mathfrak {c}_X^{-1}h(\bar{x})} \\ \le \,&  ~ (1 + \mathfrak {c}_X^{-1})\textrm{SLip}(f, A) \varepsilon _n + \delta . \end{matrix}} \end{aligned}$$

Combining (2.29), (2.30) and (2.31), we directly get the desired estimate and (2.26) is established.

Inequality (2.27) follows with similar arguments and is left to the reader.

Finally, to see that $\bar{u}$ is an AMSL extension of *f*, fix $\delta >0$ and $V \in \mathcal {P}(X\!\setminus \!A)$ and take the limit as $n\rightarrow \infty$ to deduce:

$$ \min _{y \in \partial V} \{ \bar{u}(y) + \textrm{SLip}(\bar{u}, \partial V) d^+(y, x) \} + 2\delta \ge \bar{u}(x) \ge \max _{y \in \partial V} \{ \bar{u}(y) - \textrm{SLip}(\bar{u}, \partial V) d^+(x, y) \} - 2\delta . $$

Since $\delta >0$ is arbitrary, the result follows. $\square$

## Appendix

### Proof of Theorem 2.4

Let us first state a principle of *comparison with cones* for AMSL extensions (Proposition 3.1). This characterization together with an adaptation of Perron method will be used to establish the existence of AMSL extension under given boundary data $f \in \textrm{SLip}(A)$ , where *A* is a nonempty closed subset of *X* (Theorem 2.4). Roughly speaking, the Perron method says that the supremum of every minimal semi-Lipschitz extension of *f* satisfying (P1) of Proposition 3.1 is a natural candidate for being an AMSL extension.

#### Proposition 3.1

Let $(X, d^+)$ be a convex quasi–metric space and $U \subset X$ be a nonempty open set. Then, $u: U \rightarrow \mathbb {R}$ is AMSL with respect to $\mathcal {P}(U)$ if and only if for every $V \in \mathcal {P}(U)$, it holds

$$\mathrm {(P1)}$$: $$u(x) \le \Psi (u|_{\partial V})(x)$$ for all $$x \in V$$;
$$\mathrm {(P2)}$$: $$u(x) \ge \Lambda (u|_{\partial V})(x)$$ for all $$x \in V$$.

#### Proof

($\Rightarrow$) Assume that $u \in \text {AMSL}(U)$ and $V \in \mathcal {P}(U)$. Thanks to the definition of AMSL and the continuity of *u*, it holds $\textrm{SLip}(u, \overline{V}) = \textrm{SLip}(u, \partial V)$. This says that $u \vert _{\overline{V}}$ is a minimal semi–Lipschitz extension of $u \vert _{\partial V}$. Thanks to the extremality of $\Psi (u|_{\partial V})$ and $\Lambda (u|_{\partial V})$, we get

$$\begin{aligned} \Lambda (u|_{\partial V})(x) \le u(x) \le \Psi (u|_{\partial V})(x), \quad \text { for all } x \in V. \end{aligned}$$

($\Leftarrow$) Let us now assume that *u* satisfies (P1)–(P2) for every $V \in \mathcal {P}(U)$. To this end, fix a nonempty open set $V \in \mathcal {P}(U)$. It follows readily from (P1)–(P2) that

3.1 $$\begin{aligned} u(z) - \textrm{SLip}(u,\partial V)d^+(x,z)\le u(x)\le u(z)+ \textrm{SLip}(u,\partial V)d^+(z,x), \quad \text {for all } x\in V \text { and } z\in \partial V. \end{aligned}$$

Since *X* is a convex space, for every $x\in V$ we have

$$ x\in \partial (\underbrace{V\!\setminus \!\{x\}}_{V_1})\qquad \text {and}\qquad \partial V \subset \partial V_1. $$

Setting $V_1\,{:}{=}\,V\!\setminus \!\{x\}$ we deduce from the above inclusion that $\textrm{SLip}(u,\partial V_1)\ge \textrm{SLip}(u,\partial V)$. Taking into account inequalities (3.1) we conclude that equality holds:

$$\begin{aligned} \textrm{SLip}(u,\partial V_1) = \textrm{SLip}(u,\partial V), \quad \text { for all } x \in V. \end{aligned}$$

Let now $x,y\in V$. Repeating the above procedure for the open set $V_1{:}{=}V\!\setminus \!\{x\}$, we infer that

$$ \textrm{SLip}(u,\partial (\underbrace{V\setminus \{x,y\}}_{V_1\!\setminus \!\{y\}})) = \textrm{SLip}(u,\partial (\underbrace{V\setminus \{x\}}_{V_1})) =\textrm{SLip}(u,\partial V), \quad \text { for all }x,y\in V. $$

We deduce that $u(x)-u(y)\le \textrm{SLip}(u,\partial V)d^+(y,x)$. Since $x,y \in V$ are arbitrary points, we obtain $\textrm{SLip}(u, V)=\textrm{SLip}(u, \partial V)$, which shows that $u\in \text {AMSL}(U)$ and the proof is complete.

The following lemma shows that the set of minimal semi-Lipschitz extensions of *f* satisfying (P1) is nonempty.

#### Lemma 3.2

Let $(X, d^+)$ be a complete convex quasi–metric space with $\mathfrak {c}_X > 0$, $A \subset X$ be nonempty and closed and $f \in \textrm{SLip}(A)$. Then, the McShane–Whitney extension $\Lambda (f): X \rightarrow \mathbb {R}$ defined by

$$\begin{aligned} \Lambda (f)(x) \,{:}{=}\, \sup _{a \in A} \left\{ f(a) - \textrm{SLip}(f, A)d^+(x, a) \right\} , \quad \text { for all } x \in X, \end{aligned}$$

satisfies property $\mathrm {(P1)}$ in Proposition 3.1.

#### Proof

Let us fix $V \in \mathcal {P}(X\!\setminus \!A)$ and set

$$\begin{aligned} \varphi (x) = {\left\{ \begin{array}{ll} \Psi (\Lambda (f)|_{\partial V})(x), &  x \in V, \\ \Lambda (f)(x), &  x \not \in V. \end{array}\right. } \end{aligned}$$

Since $A \subset X \setminus V$, we first note that $\varphi = \Lambda (f) = f$ in *A*. Applying Lemma 1.7 to the case $K = A$, $g = \Lambda (f)$, $\mathcal {O} = V$ and $\ell = \Psi (\Lambda (f) |_{\partial V})$, we infer that $\varphi$ is a minimal semi–Lipschitz extension of *f* and hence $\Lambda (f) \le \varphi$ in *X*. This yields that

$$\begin{aligned} \Lambda (f)\le \Psi (\Lambda (f)|_{\partial V}) \text { in }V \end{aligned}$$

and the proof is complete.

We are now ready to show the following stability property for semi-Lipschitz functions satisfying (P1). This is a key part of the Perron method.

#### Lemma 3.3

Let $(X, d^+)$ be a complete convex quasi–metric space with $\mathfrak {c}_X > 0$ and let $U \subset X$ be a nonempty open set. Let $\bar{L} > 0$ and $\mathcal {F}$ be a nonempty family of semi–Lipschitz functions such that for any $u \in \mathcal {F}$, *u* satisfies the property $\mathrm {(P1)}$ in Proposition 3.1 for all $V \in \mathcal {P}(U)$ and $\textrm{SLip}(u, X) \le \bar{L}$ for every $u \in \mathcal {F}$. Then, the function defined by

$$ \bar{v}(x) \,{:}{=}\, \sup _{v\in \mathcal {F}} v(x), \quad \text { for all } x \in X, $$

is semi–Lipschitz and satisfies the property $\mathrm {(P1)}$ in Proposition 3.1 for every $V \in \mathcal {P}(U)$.

#### Proof

It is straightforward to verify that $\bar{v}$ is semi–Lipschitz and $\textrm{SLip}(\bar{v}, X) \le \bar{L}$. Let us show that $\bar{v}$ satisfies (P1) of Proposition 3.1. To this end, it suffices to show that $v\le \Psi (\bar{v}|_{\partial V})$ in *V* for every $v\in \mathcal {F}$ and $V\subset \mathcal {P}(U)$. Fix $v \in \mathcal {F}$ and $V \in \mathcal {P}(X)$. We need to prove that the following open set is empty:

$$\begin{aligned} D = \left\{ x\in V \,:\, v(x)>\Psi (\bar{v}|_{\partial V})(x) \right\} . \end{aligned}$$

Observe that $\partial V \cap D = \emptyset$. Indeed, if $x \in \partial V \cap D$ then $v(x) > \Psi (\bar{v}|_{\partial V})(x) = \bar{v}(x)$, which contradicts the definition of $\bar{v}$. Let us now assume towards a contradiction that $D \ne \emptyset$ and define:

$$\begin{aligned} w(x) \,{:}{=}\, {\left\{ \begin{array}{ll} \Psi (v|_{\partial D})(x), &  x \in D, \\ \Psi (\bar{v}|_{\partial V})(x), &  x \not \in D. \end{array}\right. } \end{aligned}$$

It is straightforward to see that *w* is an extension of $\bar{v} \vert _{\partial V}$, that is

3.2 $$\begin{aligned} w(x) = \Psi (\bar{v}|_{\partial V})(x) = \bar{v}(x), \quad \text { for all } x \in \partial V. \end{aligned}$$

Since *v* and $\Psi (\bar{v}|_{\partial V})$ are continuous, we deduce from the definition of *D* that

3.3 $$\begin{aligned} v(x) = \Psi (\bar{v}|_{\partial V})(x), \quad \text { for all } x \in \partial D. \end{aligned}$$

Thanks to (3.2) and (3.3), we can apply Lemma 1.7 to the case $K = \partial V$, $g = \Psi ( \bar{v} |_{\partial V})$, $\mathcal {O} = D$, $\ell = \Psi ( v |_{\partial D})$ and $\varphi = w$ to obtain the inequality

3.4 $$\begin{aligned} w(x) \le \underbrace{\Psi (\Psi ( \bar{v} |_{\partial V})|_{\partial V})(x) = \Psi (\bar{v} |_{\partial V})(x)}_{\Psi (\bar{v} |_{\partial V}) = \bar{v} \text { on } \partial V}, \quad \text { for all } x \in X. \end{aligned}$$

Using the fact that *v* satisfies property (P1) in Proposition 3.1, we obtain for every $x\in D$

$$\begin{aligned} v(x) \underbrace{\le }_{v \text { satisfies (P1)}} \Psi (v|_{\partial D})(x) \underbrace{=}_{\text {definition of } w} w(x) \underbrace{\le }_{(3.4)} \Psi (\bar{v}|_{\partial V})(x), \end{aligned}$$

which contradicts the definition of *D*.

Therefore, *D* is empty and the proof is complete.

#### Lemma 3.4

Let $(X, d^+)$ be a complete convex quasi–metric space, $\mathfrak {c}_X > 0$ and $U \subset X$ be a nonempty open set. Assume that $u: X \rightarrow \mathbb {R}$ satisfies $\mathrm {(P1)}$ in Proposition 3.1 but not $\mathrm {(P2)}$. Then, there exist a semi–Lipschitz function $\widehat{u}: X \rightarrow \mathbb {R}$ and a nonempty subset $W \subset U$ such that $\textrm{SLip}(\widehat{u}, X) = \textrm{SLip}(u, X)$ and

1. $$\widehat{u}$$ satisfies property $$\mathrm {(P1)}$$;
2. $\widehat{u}= u$ in $X \setminus W$ and $\widehat{u}> u$ in *W*.

#### Proof

Since *u* does not satisfy (P2), there exists a nonempty open set $V \in \mathcal {P}(U)$ such that the set

$$ W \,{:}{=}\, \{x\in V\,:\,u(x)<\Lambda (u|_{\partial V})(x)\}\quad \text { is nonempty and open}.$$

Set

$$\begin{aligned} \widehat{u}(x) \,{:}{=}\, {\left\{ \begin{array}{ll} \Lambda (u|_{\partial W})(x), &  x \in W, \\ u(x), &  x \not \in W. \end{array}\right. } \end{aligned}$$

Since the functions $\Lambda (u|_{\partial V})$ and *u* are continuous, we deduce from the definition of *W* that

3.5 $$\begin{aligned} \widehat{u}(z)=\Lambda (u|_{\partial V})(z)=u(z)=\Lambda (u|_{\partial W})(z), \qquad \text {for every }z\in \partial W. \end{aligned}$$

#### Claim 1

For every $x\in W$ we have $\widehat{u}(x) > u(x)$.

#### Proof of Claim 1

We consider the function:

$$\begin{aligned} \varphi (x) \,{:}{=}\, {\left\{ \begin{array}{ll} \Lambda (u|_{\partial W})(x), &  x \in W, \\ \Lambda (u|_{\partial V})(x), &  x \in V\!\setminus \!W. \end{array}\right. } \end{aligned}$$

Note that $\varphi = \Lambda ( u |_{\partial V}) = u$ on $\partial V$. Applying Lemma 1.7 to the case $K = \partial V$, $g = \Lambda ( u |_{\partial V})$, $\mathcal {O} = W$ and $\ell = \Lambda ( u |_{\partial W})$, we infer that

3.6 $$\begin{aligned} \underbrace{\Lambda ( u |_{\partial V})(x) = \Lambda (\Lambda ( u |_{\partial V}) |_{\partial V})(x)}_{\Lambda (u |_{\partial V}) = u \text { on } \partial V} \le \varphi (x), \quad \text { for all } x \in V. \end{aligned}$$

Taking in particular $x\in W$ we have:

$$ \varphi (x)=\widehat{u}(x)\ge \underbrace{\Lambda (u|_{\partial V})(x) > u(x)}_{\text {definition of }W}, $$

as asserted. This completes the proof of the claim. $\square$

It remains to prove that $\widehat{u}$ satisfies (P1) in Proposition 3.1. We argue by contradiction in assuming that there exists a nonempty open set $V_0 \in \mathcal {P}(U)$ such that

$$\begin{aligned} W_0 \,{:}{=}\, \left\{ x \in V_0: \widehat{u}(x) > \Psi (\widehat{u}|_{\partial V_0})(x) \right\} \ne \emptyset . \end{aligned}$$

Notice that since the functions $\widehat{u}$ and $\Psi (\widehat{u}|_{\partial V_0} )$ are continuous, the set $W_0$ is open. Notice further that

3.7 $$\begin{aligned} W_0\cap \partial V_0=\emptyset \end{aligned}$$

#### Claim 2

For every $x\in W_0$ we have: $\Psi (\widehat{u}|_{\partial W_0})(x) \le \Psi (\widehat{u}|_{\partial V_0})(x)$.

#### Proof of Claim 2

Consider the function

$$\begin{aligned} \phi (x) = {\left\{ \begin{array}{ll} \Psi (\widehat{u}|_{\partial W_0} )(x), &  x \in W_0, \\ \Psi ( \widehat{u}|_{\partial V_0} )(x), &  x \not \in W_0. \end{array}\right. } \end{aligned}$$

Notice that $\phi |_{\partial V_0} = \widehat{u}|_{\partial V_0}$. By continuity and the definition of the set $W_0$ we also have:

$$ \Big (\Psi (\widehat{u}|_{\partial W_0} )(z)=\!\Big )\,\,\widehat{u}(z)=\Psi (\widehat{u}|_{\partial V_0} )(z), \qquad \text {for every } z\in \partial W_0. $$

Hence, we can apply Lemma 1.7 to the case $K = \partial V_0$, $g = \Psi ( \widehat{u} |_{\partial V_0})$, $\mathcal {O} = W_0$, $\ell = \Psi ( \widehat{u} |_{\partial W_0})$ and $\varphi = \phi$ to get that $\phi$ is a minimal semi–Lipschitz extension of $\Psi ( \widehat{u} |_{\partial V_0}) |_{\partial V_0} = \widehat{u} |_{\partial V_0}$. Therefore $\phi \le \Psi ( \widehat{u}|_{\partial V_0} )$ and consequently $\Psi ( \widehat{u}|_{\partial W_0} ) \le \Psi ( \widehat{u}|_{\partial V_0} )$ in $W_0$. $\square$

#### Claim 3

We have: $$W_0 \subset W$$.

#### Proof of Claim 3

Consider the open set

3.8 $$\begin{aligned} D \,{:}{=}\, \left\{ x \in W_0 : u(x) > \Psi ( \widehat{u}|_{\partial W_0})(x) \right\} . \end{aligned}$$

We now show that $D = \emptyset$. Indeed, if *D* is nonempty, then so is $\partial D$ (since *X* is connected) and we set

$$\begin{aligned} \psi (x) \,{:}{=}\, {\left\{ \begin{array}{ll} \Psi ( u|_{\partial D} )(x), &  x \in D, \\ \Psi ( \widehat{u}|_{\partial W_0})(x), &  x \not \in D. \end{array}\right. } \end{aligned}$$

Since the functions *u* and $\Psi ( \widehat{u}|_{\partial W_0})$ are continuous, we deduce from the definition of *D* in (3.8) that

3.9 $$\begin{aligned} \Big (\Psi ( u|_{\partial D})(z)=\!\Big )\,\, u(z)=\Psi (\widehat{u}|_{\partial W_0})(z),\quad \text { for every } z\in \partial D. \end{aligned}$$

Moreover, it is straightforward to see that $g = \widehat{u}$ on $\partial W_0$. Applying Lemma 1.7 to the case $K = \partial W_0$, $g = \Psi ( \widehat{u} |_{\partial W_0})$, $\mathcal {O} = D$, $\ell = \Psi ( u |_{\partial D})$ and $\varphi = \psi$, we conclude that $\psi$ is a minimal semi–Lipschitz extension of $\Psi ( \widehat{u} |_{\partial W_0}) |_{\partial W_0} = \widehat{u} \vert _{\partial W_0}$. Therefore, $\Psi (\widehat{u}|_{\partial W_0}) \ge \psi$ on $W_0$ and consequently, $\Psi (\widehat{u}|_{\partial W_0})(x) \ge \Psi ( u|_{\partial D})(x)= \psi (x)$ for every $x \in D$. Since *u* satisfies property (P1) and $D \in \mathcal {P}(U)$, we have

$$\begin{aligned} u(x) \le \Psi (u|_{\partial D})(x) \le \Psi (\widehat{u}|_{\partial W_0})(x), \quad \text { for all } x \in D, \end{aligned}$$

which contradicts the definition of *D*, showing that $D=\emptyset$ as asserted.

Let us now fix $x \in W_0$. We have

$$\begin{aligned} u(x) \underbrace{\le }_{D=\emptyset }\Psi ( \widehat{u}|_{\partial W_0} )(x) \underbrace{\le }_{\text {Claim 1}} \Psi ( \widehat{u}|_{\partial V_0} )(x)\underbrace{ < }_{\text {definition of }W_0}\widehat{u}(x). \end{aligned}$$

It follows that $x \in W$. $\square$

To conclude, we check that the nonemptiness of *W* leads to a contradiction. Indeed, using Lemma 3.2, Claim 2 and the fact that $\widehat{u} = \Lambda (u|_{\partial W})$ on $\partial W$, for every $x\in W_0$ we get

$$\begin{aligned} \widehat{u}(x) = \Lambda (u|_{\partial W})(x) \le \Psi \left( \Lambda (u|_{\partial W})|_{\partial W_0}\right) (x) = \Psi (\widehat{u}|_{\partial W_0})(x) \le \Psi (\widehat{u}|_{\partial V_0})(x), \end{aligned}$$

which contradicts the definition of $W_0$. This completes the proof. $\square$

We can finally conclude the proof of Theorem 2.4 (announced in Subsection 2.1).

#### Proof of Theorem 2.4

Let $\mathcal {F}$ the family of all minimal semi–Lipschitz extensions of $f \in \textrm{SLip}(A)$ satisfying property (P1) in Proposition 3.1 in $X\!\setminus \!A$. Since $\Lambda (f) \in \mathcal {F}$ (*c.f.* Lemma 3.2), we have $\mathcal {F} \ne \emptyset$. Furthermore, from the extremality property of the McShane–Whitney extensions (1.9), we have

3.10 $$\begin{aligned} \Lambda (f) \le v \le \Psi (f)\quad \text { and } \quad \textrm{SLip}(v, X) = \textrm{SLip}(f, A), \text { for all } v \in \mathcal {F}. \end{aligned}$$

Set

$$u(x) = \textstyle \sup _{v \in \mathcal {F}} v(x), \qquad \text { for all }x \in X.$$

We deduce easily from (3.10) that *u* is also a minimal semi–Lipschitz extension of *f*. Applying Lemma 3.3, the function *u* satisfies property (P1) in $X\!\setminus \!A$. Assume that *u* fails to satisfy property (P2). We immediately get a contradiction because of the existence of $\widehat{u}$ in Lemma 3.4 and the definition of *u*.

To conclude, since the function *u* satisfies both property (P1) and (P2), we get $u \in \text {AMSL}(X\!\setminus \!A)$ by applying Proposition 3.1. Theorem 2.4 is proven.

### A discussion on convex quasi-metric spaces

In this section, we give a short discussion on the (metrically) convex structure in the asymmetric setting. (We recall that all topological notions refer to the symmetric topology $\tau ^s$.)

#### Lemma 3.5

(convexity vs connectedness) Let $(X, d^+)$ be complete convex and $\mathfrak {c}_X > 0$.

(*i*). For every open set $\mathcal {O}\!\subset \!X$, $a \in \mathcal {O}$ and $b \in X\!\setminus \!\mathcal {O}$, it holds $[a, b] \cap \partial \mathcal {O} \ne \emptyset$.

(*ii*). The space *X* is connected.

#### Proof

(i). Let $\mathcal {O}\!\subset \!X$ be an open set. Since the assertion holds vacuously if either $\mathcal {O}=\emptyset$ or $\mathcal {O}=X$, we may assume that both $\mathcal {O}$ and $X\!\setminus \!\mathcal {O}$ are nonempty. Fix $a \in \mathcal {O}$ and $b \in X\!\setminus \!\mathcal {O}$ and set

$$\begin{aligned} \Lambda \,{:}{=}\, [a, b] \cap (X \setminus \mathcal {O}). \end{aligned}$$

Note that $\Lambda$ is closed (since for every $z \in X$ the functions $d^+(\cdot , z)$ and $d^+(z, \cdot )$ are $\tau ^s$–continuous).

We define the following binary relation on $\Lambda$:

$$ x \preceq y \quad \Longleftrightarrow \quad y \in [x, b] \cap (X \setminus \mathcal {O}). $$

It is straightforward to check that $(\Lambda , \preceq )$ is a partially ordered set.

*Claim.* Every chain in $(\Lambda , \preceq )$ has a lower bound.

#### Proof of the claim

Let $C = \{ \omega _i \}_i$ be a chain in $\Lambda$. Denote $r_0 = \inf _{\omega _i \in C} d^+(a, \omega _i) \ge 0$. If the infimum is attained at some $\bar{\omega } \in C$, then $\bar{\omega }$ is a lower bound. If the infimum is not attained, then we construct, using induction, a sequence $\{\omega _{i_n}\}_{n}\subset C$ such that $\{d^+(a, \omega _{i_n})\}_{n}$ is decreasingly convergent to $r_0$. Let now $n,m\in \mathbb {N}$ and assume, with no loss of generality, that $n\ge m$. It follows that $\omega _{i_n} \preceq \omega _{i_m}$. Hence, since $\mathfrak {c}_X > 0$, we get

$$\begin{aligned} \mathfrak {c}_X d^s(\omega _{i_n}, \omega _{i_m}) \le d^+(\omega _{i_n}, \omega _{i_m}) \underbrace{=}_{\omega _{i_n}\in [a,\omega _{i_m}]} d^+(a, \omega _{i_m}) - d^+(a, \omega _{i_n}). \end{aligned}$$

The above easily yields that the sequence $\{ \omega _{i_n} \}_{n \in \mathbb {N}}$ is $d^s$-Cauchy in *X*. Since *X* is complete, there exists $\bar{\omega }\in X$ such that $\omega _{i_n} \rightarrow \bar{\omega }$ as $n \rightarrow \infty$. Furthermore, since $\Lambda$ is closed, we obtain that $\bar{\omega }\in \Lambda$.

We shall prove that $\bar{\omega }$ is a lower bound of *C* with respect to the relation $\preceq$. Indeed, let us fix $\omega _i \in C$ and observe that $d^+(a, \omega _i) \ge d^+(a, \omega _{i_n})$ for all $n \ge N$ sufficiently large. As a consequence, we have $\omega _{i_n} \preceq \omega _i$ and consequently

$$ d^+(\omega _{i_n}, \omega _i) + d^+(\omega _i, b) = d^+(\omega _{i_n}, b), \quad \text { for all } n \ge N. $$

Letting $n \rightarrow \infty$, we infer that

$$ d^+(\bar{\omega }, \omega _i) + d^+(\omega _i, b) = d^+(\bar{\omega }, b). $$

We have shown that $\bar{\omega }\preceq \omega _i$ and since $\omega _i$ is arbitrary in *C*, the claim is proved. $\square$

Therefore, we can apply Zorn lemma to deduce that $(\Lambda , \preceq )$ contains at least one minimal element, which is denoted by $\bar{x}$. Notice that

3.11 $$\begin{aligned} d^+(a, \bar{x}) = \min _{x \in \Lambda } d^+(a, x). \end{aligned}$$

To prove that $\bar{x} \in \partial \mathcal {O}$, it suffices to check that $\bar{x} \in \overline{\mathcal {O}}$. Indeed, if $\bar{x} \notin \overline{\mathcal {O}}$, then using the fact that the topologies $\tau ^s$, $\tau ^+$ and $\tau ^-$ coincide (thanks to the assumption $\mathfrak {c}_X > 0$), there would exist $r\in \left( 0, d^+(a, \bar{x})\right)$ sufficently small such that $B^-(\bar{x}, r) \cap \mathcal {O} = \emptyset$. Since $(X, d^+)$ is a convex space, there exists $z \in X$ such that

3.12 $$\begin{aligned} d^+(z, \bar{x}) = r/2 \quad \text { and } \quad d^+(a, z) + d^+(z, \bar{x}) = d^+(a, \bar{x}). \end{aligned}$$

Observe that $z \in B^-(\bar{x}, r) \subset X \setminus \mathcal {O}$. Moreover, using the second identity in (3.12) and the fact that $\bar{x} \in [a, b]$, we get that $z \in [a, b]$. Hence, $z \in \Lambda$ and

$$ d^+(a, z) \underbrace{=}_{(3.12)} d^+(a, \bar{x}) - r/2 < d^+(a, \bar{x}), $$

which contradicts (3.11). It follows that $\bar{x}\in \overline{\mathcal {O}}\cap \Lambda$ and consequently $\partial \mathcal {O} \cap [a,b] \ne \emptyset$.

(ii). It follows from (i) that for every nonempty open subset $\mathcal {O}$ of *X*, such that $\mathcal {O}\ne X$ we have $\partial \mathcal {O} \ne \emptyset$. This yields that $\overline{\mathcal {O}} \ne \mathcal {O}$ or equivalently that $X\!\setminus \mathcal {O}$ is not open and consequently *X* cannot be written as disjoint union of two open set in a nontrivial way. The proof is complete.

#### Remark 3.6

(related notions to convexity) Based on the literature on (symmetric) metric spaces, one can also define the notion of *metrically convex* quasi–metric space as follows:

(MC)    for every $x, y \in X$ there exists $z \in X \setminus \{x, y\}$ such that $d^+(x, z) + d^+(z, y) = d^+(x, y)$.

It is clear that this notion is weaker than the notion of a convex quasimetrix space (*c.f.* Definition 1.3). However, if the space *X* is complete and $\mathfrak {c}_X > 0$, then it turns out that the two notions are equivalent. In particular, one can prove, in the spirit of Lemma 3.5, that if $(X, d^+)$ is metrically convex, complete and $\mathfrak {c}_X > 0$, then the following property (G) holds:

$\quad \mathrm {(G)}$   for every $x, y \in X$, there exists a continuous curve  $\gamma : [0, d^+(x, y)] \rightarrow X$ joining *x* and *y*

such that    $d^+(x, \gamma (t)) + d^+(\gamma (t), y) = d^+(x, y)$,   for all   $t \in [0, d^+(x, y)]$.

We can call a quasi-metric space satisfying the above property (G) *geodesic quasi–metric space*. Notice that (G) clearly yields Definition 1.3 and also connectedness of the space $(X, d^+)$. Therefore, if $(X,d^+)$ is complete and $\mathfrak {c}_X > 0$, then the properties of *X* being convex (Definition 1.3), metrically convex (MC) and geodesic (G) coincide.

We shall now show that the property that a quasi-metric space is convex is maintained if we consider the reverse metric.

#### Lemma 3.7

A quasi–metric space $(X, d^+)$ is convex if and only if the space $(X, d^-)$ is convex.

#### Proof

Let us first assume that $(X, d^+)$ is a convex quasi-metric space. We shall prove that $(X, d^-)$ is also a convex quasi-metric space. Fix $x, y \in X$ and $0< r < d^-(x, y) = d^+(y, x)$. Since $(X, d^+)$ is convex, there exists $z \in X$ such that

$$\begin{aligned} d^+(y, z) = d^+(y, x) - r \quad \text { and } \quad d^+(y, z) + d^+(z, x) = d^+(y, x). \end{aligned}$$

The above identies clearly imply that

$$ d^-(x, z) + d^-(z, y) = d^-(x, y) $$

$$ \text { and } \qquad d^-(x, z) = d^+(z, x) = d^+(y, x) - d^+(y, z) = r. $$

The inverse implication can be proved analogously.

The following examples illustrate the difference of being convex space between a quasi-metric space $(X, d^+)$ and its symmetrization $(X, d^s)$.

#### Example 3.8

Consider $X = \mathbb {R}$ and define

$$\begin{aligned} d^+(x, y) \,{:}{=}\, {\left\{ \begin{array}{ll} y - x, &  \text { if } y \ge x, \\ x - y, &  \text { if } x - 1 \le y< x, \\ \sqrt{x - y}, &  \text { if } y < x - 1. \end{array}\right. } \end{aligned}$$

We can see that $(\mathbb {R}, d^+)$ is a quasi–metric space and it is not a convex space. With direct computations, we get $d^s(x, y) = |x - y|$ and we have $(\mathbb {R}, d^s)$ is a convex space.

#### Example 3.9

Consider $X = [1, 3]$ and define

$$\begin{aligned} d^+(s, t) \,{:}{=}\, {\left\{ \begin{array}{ll} 4 \max \{ s - t, 0 \} + \max \{ t - s, 0 \}, &  \text { if } 1 \le s, t \le 2, \\ \max \{ s - t, 0 \} + 3 \max \{ t - s, 0 \}, &  \text { if } 2 \le s, t \le 3, \\ d^+(s, 2) + d^+(2, t), &  \text { otherwise.} \end{array}\right. } \end{aligned}$$

One can check that $(X, d^+)$ is a convex quasi–metric space. However, $(X, d^s)$ is not a convex space.

## Data Availability

The manuscript has no associated data.
